# P-glycoprotein and breast cancer resistance protein in acute myeloid leukaemia cells treated with the Aurora-B Kinase Inhibitor barasertib-hQPA

**DOI:** 10.1186/1471-2407-11-254

**Published:** 2011-06-16

**Authors:** Martin Grundy, Claire Seedhouse, Nigel H Russell, Monica Pallis

**Affiliations:** 1 Department of Academic Haematology, The University of Nottingham, Clinical Sciences Building, Hucknall Road, Nottingham, NG5 1PB, UK; 2 Department of Academic Haematology, Nottingham University Hospitals (City Campus), Clinical Sciences Building, Hucknall Road, Nottingham, NG5 1PB, UK; 3 Centre for Clinical Haematology, Nottingham University Hospital (City Campus), Hucknall Road, Nottingham, NG5 1PB, UK

## Abstract

**Background:**

Aurora kinases play an essential role in orchestrating chromosome alignment, segregation and cytokinesis during mitotic progression, with both aurora-A and B frequently over-expressed in a variety of human malignancies. Over-expression of the ABC drug transporter proteins P-glycoprotein (Pgp) and Breast cancer resistance protein (BCRP) is a major obstacle for chemotherapy in many tumour types with Pgp conferring particularly poor prognosis in acute myeloid leukaemia (AML). Barasertib-hQPA is a highly selective inhibitor of aurora-B kinase that has shown tumouricidal activity against a range tumour cell lines including those of leukaemic AML origin.

**Methods:**

Effect of barasertib-hQPA on the pHH3 biomarker and cell viability was measured in a panel of leukaemic cell lines and 37 primary AML samples by flow cytometry. Pgp status was determined by flow cytometry and BCRP status by flow cytometry and real-time PCR.

**Results:**

In this study we report the creation of the cell line OCI-AML3DNR, which over-expresses Pgp but not BCRP or multidrug resistance-associated protein (MRP), through prolonged treatment of OCI-AML3 cells with daunorubicin. We demonstrate that Pgp (OCI-AML3DNR and KG-1a) and BCRP (OCI-AML6.2) expressing AML cell lines are less sensitive to barasertib-hQPA induced pHH3 inhibition and subsequent loss of viability compared to transporter negative cell lines. We also show that barasertib-hQPA resistance in these cell lines can be reversed using known Pgp and BCRP inhibitors. We report that barasertib-hQPA is not an inhibitor of Pgp or BCRP, but by using ^14^[C]-barasertib-hQPA that it is effluxed by these transporters. Using phosphoHistone H3 (pHH3) as a biomarker of barasertib-hQPA responsiveness in primary AML blasts we determined that Pgp and BCRP positive primary samples were less sensitive to barasertib-hQPA induced pHH3 inhibition (p = <0.001) than samples without these transporters. However, we demonstrate that IC_50 _inhibition of pHH3 by barasertib-hQPA was achieved in 94.6% of these samples after 1 hour drug treatment, in contrast to the resistance of the cell lines.

**Conclusion:**

We conclude that Pgp and BCRP status and pHH3 down-regulation in patients treated with barasertib should be monitored in order to establish whether transporter-mediated efflux is sufficient to adversely impact on the efficacy of the agent.

## Background

The mammalian aurora kinases aurora-A, aurora-B and aurora-C comprise a family of serine/threonine kinases that are essential for cell cycle control and mitotic progression [1]. Interest in the auroras has intensified since the observation that both aurora-A and B are over-expressed in a wide variety of tumour types [2-5] including those of leukaemic origin [6,7]. The implication of the auroras in tumourigenesis and the fact that that they are kinases, amenable to small molecule inhibition, makes them attractive targets for anticancer drug development. Success of agents such as imatinib in the treatment of chronic myelogenous leukaemia has increased confidence that small-molecule inhibitors of specific kinases may prove to be highly effective anticancer agents [8]. Despite having high sequence homologies in their kinase domains the three aurora members have very distinct subcellular localizations and functions during mitosis [9]. Aurora-B is a chromosomal passenger protein which undergoes dynamic localization during mitosis, associating first to the inner centromeric region during prometaphase, and then to the spindle midzone and midbody during late anaphase and telophase suggesting a role in cytokinesis [1,10]. Aurora-B is the catalytic component of the chromosomal passenger complex (CPC), which is composed of three additional non-catalytic subunits that direct its activity: survivin, inner centromere protein (INCENP) and borealin. The CPC orchestrates the spindle checkpoint and ensures the accurate segregation of chromatids and correct microtubule/kinetochore attachment during mitosis and cytokinesis [11]. Aurora-B is also known to phosphorylate Histone H3 (pHH3) at the serine 10 position during mitosis [12,13]. Inhibition of Histone H3 phosphorylation has been reported to prevent initiation of chromosome condensation and entry into mitosis [14]. Aurora-A is known to phosphorylate numerous centrosomal proteins and primarily functions in centrosomal regulation and mitotic spindle formation with loss of Aurora-A function leading to cell cycle arrest and monopolar mitotic spindles [9]. Aurora-C is the least studied of the aurora family and is highly expressed in the testis where it is thought to have a specific role in the regulation of chromosome segregation during male meiosis [15]. More recently aurora-c has been identified at low levels in sixteen other tissues including bone marrow with studies suggesting that it has a complementary role to aurora B and Survivin as a chromosomal passenger protein [16,17].

A growing number of aurora kinase inhibitors have been described that show anti-tumour activity *in vivo*. Three non-selective aurora kinase inhibitors ZM447439, Hesperadin and VX-680 all induce similar phenotypes when tested in cell based assays [18-20]. Specifically, all three inhibit phosphorylation of Histone H3 on serine 10 and induce DNA endoreduplication in the absence of cytokinesis, results that suggest that their cellular effects are largely due to the inhibition of aurora-B [21]. We have previously reported the same cellular phenotype in AML cell lines treated with barasertib-hQPA [22].

Barasertib (formerly AZD1152) is a quinazoline prodrug which is converted in plasma to the more active moiety barasertib-hQPA (AZD1152-hQPA) and it is the more active barasertib-hQPA that has been supplied by AstraZeneca for the purpose of this study. Barasertib-hQPA is an aurora kinase inhibitor that has potent selectivity for inhibition of aurora-B (Ki: aurora-B = 0.36 nM) compared to aurora-A and C (K_i_: aurora-A = 1369 nM and aurora-C = 17.0 nM) and a panel of 50 other kinases [23]. We have however recently reported that the FMS-like tyrosine kinase 3 internal tandem duplication (FLT3-ITD) mutation is a secondary target for barasertib-hQPA in AML cells [22]. Barasertib has been shown to significantly inhibit the growth of human colon, lung and haematological tumour xenografts in immunodeficient mice and as such has been selected for clinical evaluation [23,24]. It has also shown tumouricidal activity against a panel of tumour cell lines including those of acute myeloid leukaemia (AML) origin [22,25-27]. Results of a Phase 1 study in AML were reported at ASH 2010 (Abstract 656) and a Phase II study is ongoing.

AML is a heterogeneous clonal disorder of haemopoietic progenitor cells where both failure to differentiate and over proliferation results in accumulation of non-functional cells termed myeloblasts [28]. Intrinsic resistance or treatment-induced acquired resistance is one of the major obstacles to the effective treatment of patients with AML. While nearly 80% of younger AML patients may initially achieve complete remission with current therapy most will relapse with resistant disease [29]. Clinical outcomes in the elderly have been even more modest as these patients do not tend to tolerate intensive chemotherapy regimens and frequently have poor cytogenetics [30]. Less than 10% of older patients with AML will achieve long-term disease free survival with conventional chemotherapy [31]. This inability to successfully treat AML patients, particularly the elderly, underlies the continuing need to develop new treatments for AML.

The development of multidrug resistance (MDR) is frequently observed in the treatment of cancer, and is a phenomenon that allows tumour cells that have been exposed to one cytotoxic agent to develop cross resistance to a range of structurally and functionally unrelated compounds. In patients with AML, MDR can be present intrinsically at diagnosis or can arise during chemotherapy as well as at relapse. ATP binding cassette (ABC) transporter-mediated active efflux of cytotoxic agents is the most well characterized mechanism by which cancer cells develop MDR, particularly after repeated cycles of chemotherapy [32]. The ABC transporters are an evolutionary extremely well conserved family of transmembrane proteins expressed in most cells and involved in the ATP driven transport of a huge variety of substrates including sugars, peptides, inorganic ions, amino acids, proteins, vitamins and metallic ions [33]. The family currently consists of 49 members, 13 of which are associated with chemotherapeutical drug transport and drug resistance [34]. The genes can be divided into subfamilies based on similarity in gene structure resulting in seven mammalian ABC gene subfamilies (ABCA, B C, D, E F and G family). The most widely studied members ABCB1 (MDR1/P-glycoprotein), ABCC1 (multidrug resistance protein, MRP1) and ABCG2 (breast cancer resistance protein, BCRP) have the ability to export a wide variety of structurally unrelated chemotherapeutic compounds from cancer cells, thereby conferring MDR to these cell [33].

Despite improvements accomplished in the last thirty years with the use of combination of cytarabine and intercalating agents, the overall prognosis of adult AML remains poor. One of the best characterized resistance mechanisms in AML is drug extrusion mediated by P-glycoprotein (Pgp). Expression of the MDR1 gene coding for Pgp is high in elderly patients with AML and is associated with worse complete remission rates [30]. Multidrug resistance-associated protein 1 (MRP1) has also been shown to contribute to MDR in AML [30], whereas other studies have been unable to detect any correlation between MRP1 protein expression and clinical response in AML patients [35]. Breast cancer resistance protein (BCRP) has been shown to be overexpressed in 33% of AML patients with normal karyotype and to significantly affect the duration of complete remissions [36]. Another study showed BCRP expression to be a prognostic factor in AML patients treated with daunorubicin and mitoxantrone but not with idarubicin [37]. Conversely, another group found a prognostic value for Pgp and MRP1 but suggested BCRP has a limited function in the drug efflux related resistance in AML [38]. It has recently been reported that the amplification of genes that encode for Pgp and BCRP can confer resistance to barasertib-hQPA in colon and pancreatic carcinoma cell lines [39].

In this study we will investigate the specificity of barasertib-hQPA in AML cell lines and primary samples in relation to their ABC transporter status.

## Results

### Cell line validation

The OCI-AML3DNR cell line was created as described in the Methods section. The short tandem repeats (STR) analysis of the OCI-AML3DNR and OCI-AML6.2 cell lines was shown to be identical to the parent OCI-AML3 cell line indicating that there were no significant genetic changes.

### Pgp and BCRP expressing cell lines are less sensitive to barasertib-hQPA induced pHH3 inhibition and subsequent loss of viability

**Figure 1:**
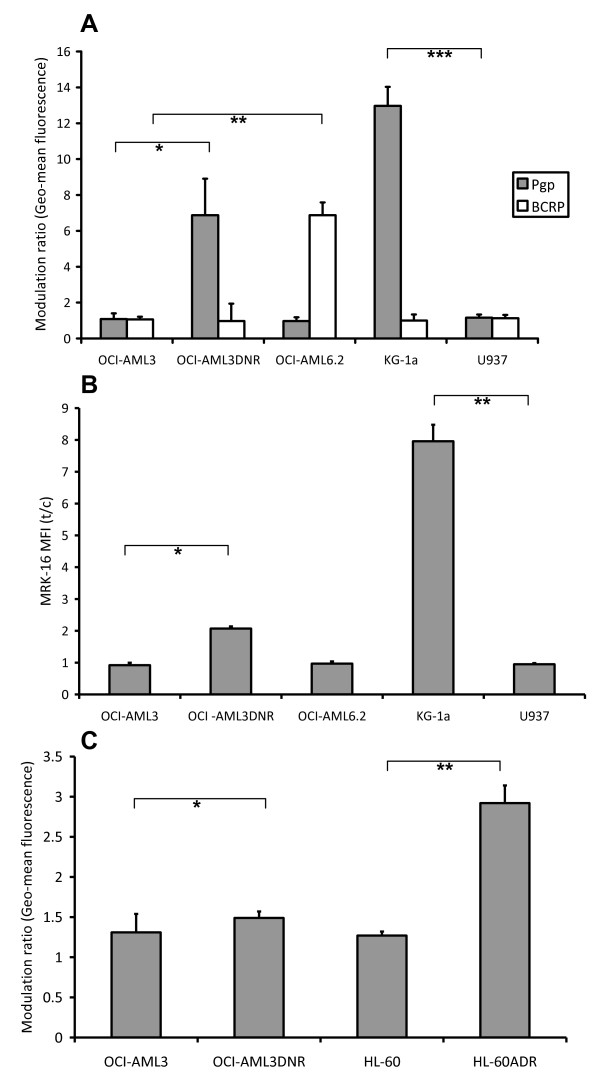
**Expression of ABC transporters in AML cell lines**. A, Pgp function was measured using R123 accumulation and Cyclosporine A as a positive control modulator. BCRP was measured similarly with BODIPY-prazosin accumulation and Fumitremorgin C as the modulator. (*p = 0.039/**p = 0.015/***p = 0.002) (analysed using paired samples t-test), +95% confidence interval). B, Pgp protein expression was determined using the MRK-16 monoclonal antibody (*p = 0.003/**p = 0.006). C, MRP function was measured using Calcein-AM accumulation and MK-571 as a modulator (*p = 0.358/**p = 0.004). Columns, mean of three experiments; bars, SD.

**Figure 2:**
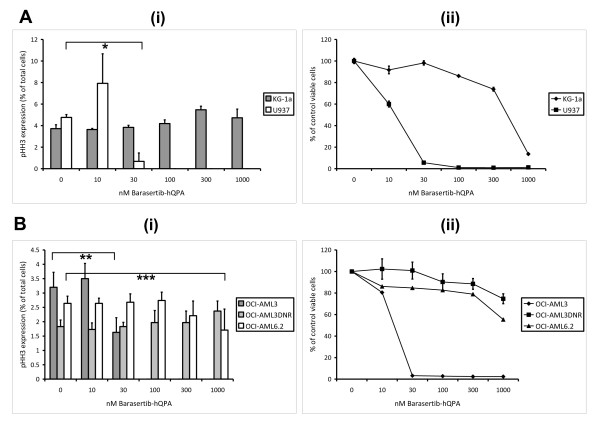
**Specificity of barasertib-hQPA in AML cell lines**. A(i), B(i) Effect of 24 hours barasertib-hQPA exposure on pHH3 expression in leukaemic cell lines (*p = 0.001/**p = 0.003/***p = 0.207) (analysed using paired samples t-test), +95% confidence interval). A(ii), B(ii) Effect of 72 hours barasertib-hQPA exposure on viable cell count in leukaemic cell lines measured using 7AAD and fixed stained cells. Columns/lines, mean of three experiments; bars, SD.

The effects of barasertib-hQPA were examined in logarithmically growing OCI-AML3, OCI-AML3DNR, OCI-AML6.2, KG-1a and U937 leukaemic cell lines. Functional Pgp was determined using rhodamine 123 (R123) retention and the known Pgp modulator cyclosporine A (CSA) as a positive control modulator [40], with the KG-1a (p = 0.002) and daunorubicin selected OCI-AML3DNR (p = 0.039) cell lines showing high Pgp functional expression (Figure 1A). Functional BCRP was measured using BODIPY-prazosin (BODIPY) retention and Fumitremorgin C (FTC) [41] as a modulator with only the stably transfected BCRP OCI-AML6.2 (p = 0.015) cell line showing any functional BCRP activity. High OCI-AML3DNR (p = 0.003) and KG-1a (p = 0.006) Pgp levels were also observed when we measured Pgp protein using the MRK-16 monoclonal antibody (Figure 1B). To verify that MRP expression had not also been elevated in our daunorubicin selected cell line we used a Calcein-AM retention assay with the MRP modulator MK-571 [42] and compared MRP function to the adriamycin selected, MRP positive, HL-60ADR cell line [43] (Figure 1C). No significant increase in MRP function was detected in the OCI-AML3DNR cells (p = 0.358) in contrast to that seen in the HL-60ADR cells (p = 0.004) when compared to the parent cell lines. An in house flow cytometry protocol for measuring pHH3 expression was used and has been described in detail previously [22]. The basal range of pHH3 expression in the cell lines was 1.8-4.8% of total cells. After 24 hours, at concentrations of 0-1000 nM barasertib-hQPA, inhibition of pHH3 was achieved in both ABC transporter negative cell lines (Figure 2A(i) and 2B(i)). There was significant inhibition of pHH3 in the U937 (p = 0.001) and OCI-AML3 (p = 0.003) cells at 30 nM barasertib-hQPA before complete inhibition at 100 nM. The ABC transporter positive cell lines were all much more resistant to pHH3 inhibition at barasertib-hQPA concentrations up to 1000 nM. The decrease in pHH3 seen at 300 nM and 1000 nM barasertib-hQPA in the OCI-AML6.2 cells was not statistically significant (p = 0.408/0.207). Seventy-two hours incubation with barasertib-hQPA caused loss of viability in both transporter negative cell lines with an almost complete loss of viability achieved at 30 nM (Figure 2A(ii) and 2B(ii)). Again, the transporter positive cells were much more resistant, with loss of viability seen only at high dose barasertib-hQPA.

### Barasertib-hQPA is not an inhibitor of Pgp or BCRP but it is effluxed by these transporters

**Figure 3:**
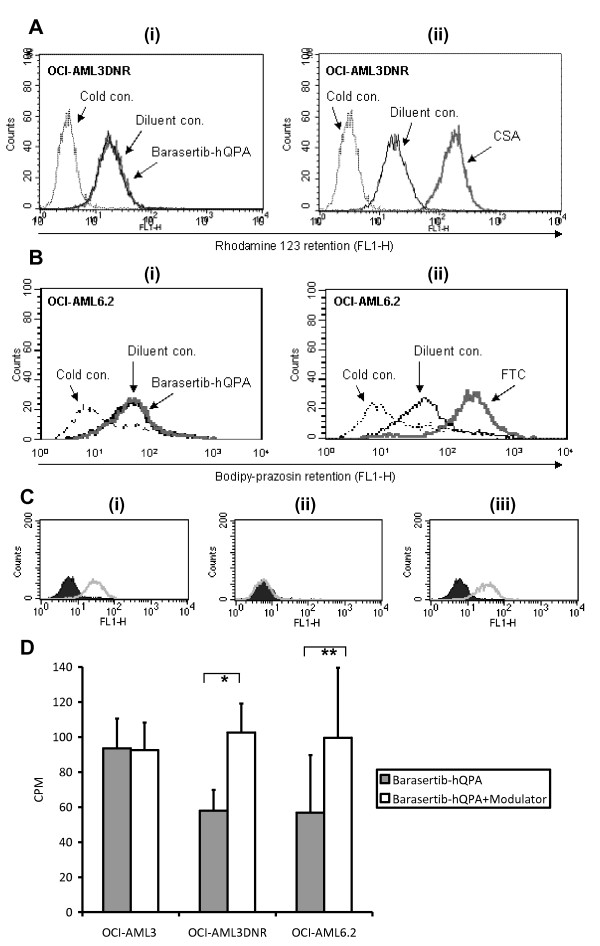
**There is no modulation of Pgp or BCRP function by barasertib-hQPA**. A, R123 accumulation in OCI-AML3DNR cells +/- 300 nM barasertib-hQPA (i) or 2.5 μg/ml CSA (ii). The dotted lines are the 4°C R123 control. The solid black line is R123 + diluent control and the solid grey line R123 + barasertib-hQPA (i) and R123 + CSA (ii). B, BODIPY-prazosin accumulation in OCI AML6.2 cells +/- 300 nM barasertib-hQPA (i) or 10 μM FTC (ii). The dotted lines are the 4°C BODIPY control. The solid black line is BODIPY + diluent control and the solid grey line BODIPY + barasertib-hQPA (i) and BODIPY + FTC (ii). Plots are representative of a single experiment which was done in triplicate. C, UIC2 binding in KG-1a cells in the presence of 5 μM vinblastine (i), 1 μM barasertib-hQPA (ii) or a combination of both (iii). The dark filled line is the diluent control and the light line indicates any shift. D, 300nM [^14^C]-barasertib-hQPA uptake in Pgp expressing OCI-AML3DNR cells +/- the Pgp modulator CSA and in OCI-AML6.2 cells +/- the BCRP modulator FTC. Pgp/BCRP negative OCI-AML3 cells have been used as a positive control (*p = 0.004/**p = 0.005) (analysed using paired samples t-test), +95% confidence interval). Diluent controls were included for each modulator. Columns, mean of three experiments; bars, SD.

To determine whether barasertib-hQPA is able to modulate Pgp or BCRP function we compared it to known modulators using fluorescent dye retention assays. Barasertib-hQPA had no effect on R123 retention in the Pgp positive OCI-AML3DNR cell line (Figure 3A(i)) in contrast to the marked increase in retention seen with CSA (Figure 3A(ii)). Likewise barasertib-hQPA had no effect on BODIPY retention in the BCRP positive OCI-AML6.2 cell line (Figure 3B(i)) in contrast to the increase in retention seen with FTC (Figure 3B(ii)). In these assays barasertib-hQPA did not appear to be a modulator of Pgp or BCRP. We therefore decided to employ the UIC2 shift assay; which is an indirect way of measuring unlabelled Pgp substrates [44]. The reactivity of the UIC2 antibody with Pgp is increased by the addition of Pgp transported compounds. We tested barasertib-hQPA in the UIC2 shift assay using the Pgp positive KG-1a cell line and the known Pgp substrate vinblastine [44] as a positive control (Figure 3C). No shift in UIC2 binding was seen with barasertib-hQPA treatment. Also, co-incubation of barasertib-hQPA and vinblastine, failed to affect any shift seen with vinblastine alone. This assay failed to show that barasertib-hQPA is a Pgp substrate. However it did not prove the converse as a few Pgp substrates such as etoposide fail to alter UIC2 binding depending on their stoichiometry and Pgp ATPase activity [45]. To categorically determine if barasertib-hQPA was being effluxed by Pgp and BCRP we used radio-labelled barasertib-hQPA to measure its cellular retention (Figure 3D). In this assay CSA and FTC increased retention of [^14^C]-barasertib-hQPA in both OCI-AMLDNR (p = 0.004) and OCI-AML6.2 (p = 0.005) cells respectively. The modulators increased retention of [^14^C]-barasertib-hQPA to at least the concentration seen in the ABC transporter negative/barasertib-hQPA sensitive OCI-AML3 cell line.

### Culture with known inhibitors sensitize Pgp and BCRP positive AML cells to barasertib-hQPA

**Figure 4:**
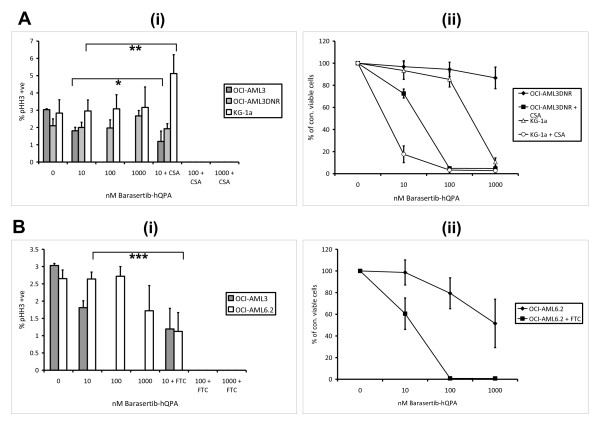
**Specificity of barasertib-hQPA in AML cell lines in combination with ABC transporter modulators**. A, Effect of 24 hours barasertib-hQPA exposure +/- 2.5 μg/ml CSA on pHH3 expression in OCI-AML3, OCI-AML3DNR and KG-1a cells (*p = 0.145/**p = 0.145) (analysed using paired samples t-test), +95% confidence interval) (i). Effect of 72 hours barasertib-hQPA exposure +/-2.5 μg/ml CSA on viable cell count (ii). B, Effect of 24 hours barasertib-hQPA exposure +/- 5 μM FTC on pHH3 expression in OCI-AML3 and OCI-AML6.2 cells (***p = 0.021) (i). Effect of 72 hours barasertib-hQPA exposure +/- 5 μM on viable cell count (ii). Columns/lines, mean of three experiments; bars, SD.

Sub-toxic doses of the Pgp inhibitor CSA and also of the BCRP inhibitor FTC were added to cell culture with 10-1000 nM barasertib-hQPA (Figure 4). Addition of CSA sensitizes the Pgp positive cell lines OCI-AML3DNR and KG-1a to pHH3 down-regulation with complete loss of pHH3 seen at 24 hours with 100 nM barasertib-hQPA (Figure 4A(i)). There is no statistical significance in the decrease in pHH3 at 10 nM barasertib-hQPA plus CSA in the OCI-AML3 cells, or in the increase in pHH3 in KG-1a cells with the same treatment, both p = 0.145. The same effect is seen in 72 hour cell viability with a marked decrease in viability at 10 nM and complete loss of cell viability at 100 nM barasertib-hQPA with the addition of CSA (Figure 4A(ii)). The MRP inhibitor MK-571 did not sensitize OCI-AML3DNR cells to barasertib-hQPA induced pHH3 inhibition, or loss of viability, confirming that resistance is not due to any elevated MRP expression in these cells (Data not shown). Addition of FTC also clearly sensitizes the BCRP positive cell line OCI-AML6.2 to pHH3 down-regulation at barasertib-hQPA concentrations as low as 10 nM (p = 0.021) with complete down-regulation of pHH3 seen at 100 nM barasertib-hQPA with the addition of FTC (Figure 4B(i)). Complete loss of cell viability at 72 hours was achieved at 100 nM barasertib-hQPA with the addition of FTC (Figure 4B(ii)).

### Pgp and BCRP positive primary AML samples are less sensitive to barasertib-hQPA induced pHH3 inhibition

**Figure 5:**
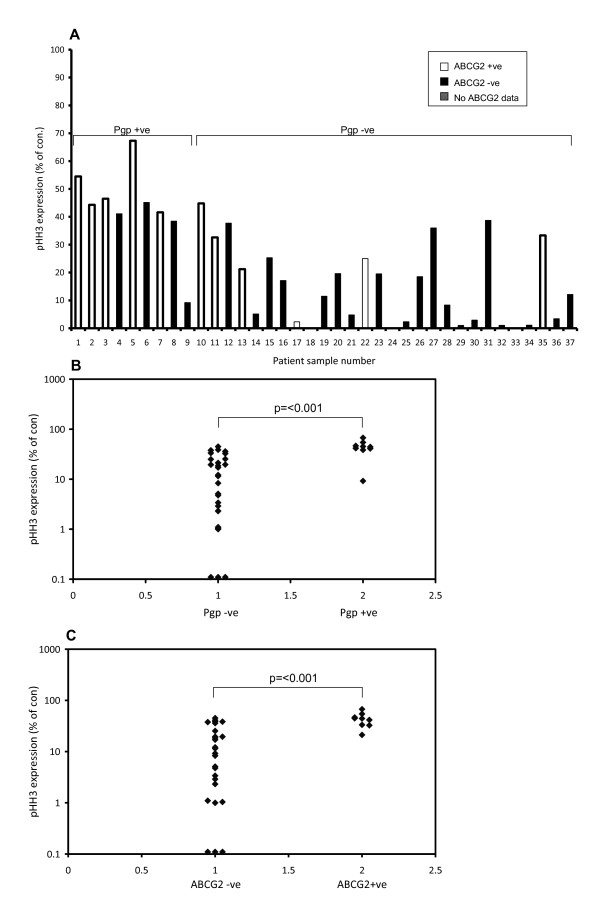
**Pgp and BCRP positive primary AML samples are less sensitive to barasertib-hQPA induced pHH3 inhibition**. A, Flow cytometric pHH3 expression in 37 primary AML samples pre-incubated with cytokine cocktail for 48 hours and then treated with 300 nM barasertib-hQPA for 60 minutes. Pgp expression was determined by modulation of R123 efflux and MRK-16 expression. ABCG2 message levels were measured using real-time PCR. B, Scatter plot demonstrating that Pgp positive primary samples are less sensitive to barasertib-hQPA induced pHH3 inhibition than Pgp negative samples (p = <0.001), Mann-Whitney. C, Scatter plot demonstrating that BCRP positive primary samples are less sensitive to barasertib-hQPA induced pHH3 inhibition than BCRP negative samples (p = <0.001), Mann-Whitney.

The level of pHH3 detectable in untreated cell lines was low (1.8-4.8% of total cells) and expression in primary cells was expected to be even lower, as a very small population of cells are actively dividing at the time of sampling. Because of this we pre-incubated primary samples with a cytokine cocktail to drive the cells into cycle before treatment with barasertib-hQPA. Cellular proliferation in primary samples has been confirmed previously using [^3^H]-Tdr uptake with pHH3 expression correlating extremely well with the amount of proliferation [22]. Mean basal pHH3 expression in the primary samples was 3.01% (range, 0.04-13.14%) of total cells. We have previously reported that basal pHH3 expression in our primary samples shows significant correlation with aurora-B mRNA levels (p = 0.015) [22]. pHH3 expression was measured in 37 primary samples after 1 hr's treatment with 300 nM barasertib-hQPA (Figure 5A). IC_50 _was achieved in all but 2 samples (94.6%) with a mean down-regulation of 78% (Range, 32.7-100%). Of the primary samples tested 9/37 (24.3%) were positive for Pgp and 9/35 (25.7%) positive for BCRP. Mean MRK-16 test/control mean fluorescence intensity (MFI) in the primary samples was 1.13 (range, 0.72-2.92). Mean CSA modulation ratio in the primary samples was 1.74 (range, 0.91-9.16). Five samples co-expressed Pgp and BCRP such that we found a significant correlation (p = 0.008, r2 = 0.238) between Pgp and BCRP expression in our primary samples. The percentage of Pgp and BCRP positive samples and the co-expression correlation agrees with previously published data [36]. Pgp positive samples were significantly (p = <0.001) less sensitive to barasertib-hQPA induced pHH3 inhibition than Pgp negative samples (Figure 5B). BCRP positive primary samples were also significantly (p = <0.001) less sensitive to barasertib-hQPA induced pHH3 inhibition compared to BCRP negative samples (Figure 5C). However, a sharp distinction between cell lines and primary samples was noted. Whereas the transporter-expressing cell lines were insensitive to down-regulation of pHH3 at concentrations of up to 1000 nM barasertib-hQPA even after 24 hours, IC_50 _pHH3 inhibition was achieved in 94.6% of primary samples at 300 nM barasertib-hQPA for one hour.

## Discussion

The success of agents such as imatinib in the treatment of chronic myelogenous leukaemia has lead to an increase in confidence that small-molecule inhibitors of specific kinases may prove to be highly effective anticancer agents [8]. The aurora kinase family are essential for normal mitotic progression and have attracted great interest as potential new therapeutic targets. Overexpression of aurora kinases have been reported in many tumour types and linked to a poor prognosis. The aurora-B kinase specific inhibitor barasertib-hQPA has shown tumouricidal activity against a panel of tumour cell lines and xenograft models including those of AML origin [22-24,27]. Our group have recently reported that the FLT3-ITD mutation is a secondary target for barasertib-hQPA and that primary AML samples with an ITD mutation in the FLT3 gene are particularly sensitive to the drug [22]. The FLT3 gene is one of the most commonly mutated genes in AML with the FLT3-ITD mutation appearing in roughly 24% of cases and being associated with a poor prognosis [46,47]. Barasertib-hQPA is therefore of potential use as a future treatment in AML.

Intrinsic MDR or treatment-induced MDR has historically been one of the major obstacles to the effective treatment of patients with AML. Elevated expression of the MDR1 gene coding for the drug efflux pump Pgp is one of the best characterized resistance mechanisms in AML with high expression in elderly patients particularly associated with worse complete remission rates [30]. The ABCG2 gene encoding BCRP has also been shown to be overexpressed in AML patients and to significantly affect the duration of complete remission [36]. Any potential new drug for the treatment of AML should therefore be screened for its efficacy in patients expressing high levels of these drug transporters.

Aurora-B is known to phosphorylate Histone H3 (pHH3) at the serine 10 position during mitosis [12,13]. We have previously demonstrated a method for measuring pHH3 expression in our cell lines and more importantly in our primary samples and its subsequent down-regulation after barasertib-hQPA treatment [22]. We can therefore use pHH3 expression as a biomarker for barasertib-hQPA activity. Initial experiments showed that our Pgp positive (KG-1a) and BCRP positive (OCI-AML6.2) cell lines were much less sensitive to barasertib-hQPA in comparison to transporter negative cell lines. This was seen at both the biomarker and subsequent viability level. Because we had no parental Pgp negative comparison for the KG-1a cell line we wanted to create a Pgp expressing cell line from the OCI-AML3 cells. The subsequently developed Pgp expressing OCI-AML3DNR cell line was more resistant to barasertib-hQPA compared to the sensitive OCI-AML3 parent cells (Figure 2B). This resistance to barasertib-hQPA in the transporter positive cell lines was reversed when known inhibitors of Pgp and BCRP were co-cultured with barasertib-hQPA (Figure 4). There was an initial spike in KG-1a pHH3 levels at 10 nM barasertib-hQPA with the addition of CSA (Figure 4A(i)). This spike was also seen in the sensitive U937 cells also at 10 nM barasertib-hQPA (Figure 2A(i)) and to a lesser extent in the OCI-AML3 cells (Figure 2B(i)) indicating that CSA is sensitizing the Pgp expressing KG-1a cells to barasertib-hQPA. By using ^14^[C]-barasertib-hQPA in combination with known Pgp and BCRP inhibitors we show definitively that the drug is being effluxed by these transporters in the OCI-AML3DNR and OCI-AML6.2 cell lines.

Inhibiting Pgp as a way of reversing MDR has been intensively studied for many years. Pharmacological inhibition of Pgp by small-molecule antagonists has been studied in several AML trials with various agents but only yielding little clinical success [48-51]. Many of these agents are substrates for other transporters and enzyme systems resulting in unpredictable pharmacokinetic interactions in the presence of chemotherapy agents. Recently more specific so called third generation inhibitors of MDR have been developed that have the potential to minimize any drug-drug interactions [52]. A combination of these inhibitors with barasertib-hQPA could circumvent any problem of drug efflux by ABC transporters seen with this drug.

We measured pHH3 expression in 37 primary AML samples cultured for one hour with or without barasertib-hQPA. 9/37 (24.3%) were positive for Pgp and 9/35 (25.7%) were positive for BCRP with a significant correlation (p = 0.008, r2 = 0.238) seen for co-expression. This data agrees with results seen previously [36]. Pgp positive samples were significantly less sensitive to barasertib-hQPA induced pHH3 inhibition compared to Pgp negative samples (p = 0.001) as were BCRP positive samples compared to BCRP negative samples (p = 0.001). Importantly though, >50% inhibition of pHH3 was still seen in all but 2 primary samples (94.6%). So certainly at the level of the biomarker we see a much better response in ABC transporter positive primary samples than in transporter positive cell lines at the corresponding dose of barasertib-hQPA. Whereas in the transporter positive cell lines we demonstrated efflux of barasertib-hQPA using radio-labelled drug it is clear that all of our primary samples retained enough drug to target the pHH3 biomarker. This difference in response may be explained by the 'artificially' high transporter expression seen in the cell lines. For example, if we look at our measure of Pgp function, the mean CSA modulation ratio in our primary samples was 1.74. Four times this was seen in the Pgp expressing OCI-AML3DNR cell line and greater than seven times seen in the Pgp expressing KG-1a cell line. Likewise the amount of Pgp protein measured using the MRK-16 antibody was two-folds higher in the OCI-AML3DNR cells and seven-folds higher in the KG-1a cells compared to the mean value of 1.13 seen in primary samples.

## Conclusions

MDR AML cell lines are a useful tool to model transporter mediated drug resistance but their efflux activity is extreme compared to that found in primary AML samples. Most clinical trials with Pgp modulators have been unsuccessful and thus the importance of efflux pumps in AML chemoresistance is unclear. Clinical trials with barasertib may help to resolve this issue, since pHH3 is such a clear-cut marker of efficacy at the cellular level. We conclude that Pgp and BCRP expression levels and pHH3 down-regulation in patients treated with barasertib should be monitored in order to establish whether transporter-mediated efflux is sufficient to adversely impact on the efficacy of the agent.

## Methods

### Materials

Materials were from Sigma (Poole, Dorset UK) unless otherwise stated. Barasertib-hQPA was provided by AstraZeneca (Cheshire, UK).

### Cell lines

The OCI-AML3 myeloid leukaemia cell line was obtained from the German Collection of Microorganisms and Cell Cultures (DSMZ, Braunschweig, Germany). U937, HL-60 and KG-1a cell lines were from the European Collection of Animal Cell Cultures (Salisbury, UK). The ABCG2 stably transfected OCI-AML6.2 cells were a gift from Dr. Jo Mountford (University of Glasgow, UK). The HL-60ADR [43] cell line was a gift from Mark Center (Kansas State University, USA). U937, OCI-AML3 and OCI-AML6.2 cell lines were maintained in RPMI 1640 medium with 10% foetal calf serum (FCS; First Link, Birmingham, UK), 2 mM L-glutamine, 100 U/ml penicillin and 10 μg/ml streptomycin. The KG-1a cell line was maintained as above with 20% FCS. All cultures were kept at 37°C in 5% CO_2 _and all experiments were performed with cell lines in log phase. Continued testing to authenticate these cell lines was initially performed using a panel of monoclonal antibodies and later by STR fingerprinting. Mycoplasma testing was carried out routinely using a Mycoalert mycoplasma detection kit (Lonza, Rockland, USA) and following the manufacturer's instructions.

### Selection of Daunorubicin resistant OCI-AML3 cells

OCI-AML3 cells were initially exposed to daunorubicin (DNR) at a concentration of 10 nM, which represented the IC_50 _concentration. Cells were seeded at 5 × 10^5^/ml in 50 ml of complete medium containing 10 nM DNR. At days 3, 10 and 14, 50 ml of fresh culture medium without DNR was added. At day 18 cells were pelleted and re-suspended in 160 ml medium containing 10 nM DNR. From day 26 to day 35 the DNR containing medium was changed once per week. At day 35 there were very few remaining viable cells, so cells were separated over Histopaque to exclude dead cells and cell debris. The remaining cells were re-suspended in 10 ml of medium containing 10 nM DNR up until day 45 when cell growth was seen. Cells were maintained at this drug concentration until their growth rate approached that of untreated OCI-AML3 cells. Over the next 10 weeks cells were exposed to gradually increasing concentrations of DNR up to a final concentration of 15 nM. By using the R123 accumulation assay these cells were confirmed to have increased levels of Pgp function and were subsequently cloned by limiting dilution. Cells were serially diluted in 96 well plates down to a concentration of 1 cell/well in medium containing 15 nM DNR. Any cells showing outgrowth were then gradually cultured in medium containing 15 nM DNR up to sufficient numbers to be able to assay for Pgp protein and function. One clone showed an increase in both Pgp protein expression and function. This clone was named OCI-AML3DNR and was then cultured under standard conditions with 15 nM DNR and also cryo-preserved for future use. DNR was removed from culture medium 7 days prior to any experimental procedure.

### OCI-AML3/OCI-AML3DNR/OCI-AML6.2 genetic analysis

DNA was prepared using a QIAamp DNA blood mini kit (Qiagen, Crawley, UK) and 5 ng DNA was amplified using the Powerplex 16 System (Promega, Southampton, UK) to assess STR. The products were run on a 3130 Genetic Analyser and the data analysed using GeneMapper ID v3.2 software.

### Patient samples

Presentation blood or bone marrow samples from multiple centres were obtained at diagnosis from patients with AML (excluding M3 subtype) and taken into preservative-free heparin or into EDTA tubes. All samples were pre-treatment and only samples received within 48 hours of being removed from the patient were analyzed. Use of these samples was approved by the Nottingham 1 Research Ethics Committee (reference number 06/Q2403/16). Mononuclear cells were isolated using a standard density gradient/centrifugation method with Histopaque and cryopreserved in liquid nitrogen. For analysis, cryopreserved samples were thawed and rested in culture medium enriched to 20% FCS for 90 minutes before experimental procedures. Thawed and rested samples were then subjected to viability analysis using trypan blue exclusion and only samples with > 85% post-rest viability were used.

### Pgp protein expression - MRK-16 mAb

For Pgp protein expression cells were harvested, washed in PBSAA, and 2 × 10^5 ^cells incubated with 1 μg MRK-16 anti-Pgp monoclonal antibody (Kamiya Biomedical) or IgG2a isotype control (Dako) for 30 min at RT. Cells were washed x3 in PBSAA and blocked in 80 μl 20% normal rabbit serum for 30 min on ice. 5 μl FITC-conjugated goat anti-mouse secondary antibody (Dako) was added and cells incubated for 30 min on ice. Cells were washed twice in PBSAA and cell line data collected using a FACS calibur. Patient samples had the added step of the addition of 5 μl CD45PerCP (Becton Dickinson, UK) and 5 μl Normal Mouse serum before being washed twice in PBSAA and collected using a FACS calibur. Labelling the patient samples with CD45PerCP allowed the leukemic (CD45 low/side scatter low) cells to be gated [53].

### Determination of Pgp, BCRP and MRP function

Functional Pgp expression was determined by modulation of R123 efflux. Cells were resuspended at 1 × 10^6^/ml in culture medium and incubated (75 minutes, 37°c) with R123 (200 ng/ml) with or without 2.5 μg/ml CSA (supplied by the pharmacy of Nottingham City Hospital) or diluent control. Control tubes containing R123 were placed on ice to halt the reaction [53,54]. Functional BCRP expression was measured similarly and was determined by modulation of BODIPY (Invitrogen) efflux. Cells were resuspended at 1 × 10^6^/ml in culture medium and incubated (75 minutes, 37°c) with BODIPY (25 nM) with or without 10 μM FTC (Calbiochem) or diluent control. Control tubes containing BODIPY were placed on ice. MRP function was determined by modulation of Calcein-AM (Invitrogen) efflux [55]. Cells were resuspended at 1 × 10^6^/ml in culture medium and incubated (75 minutes, 37°c) with Calcein-AM (0.1 μM) with or without 40 μM MK-571 (Enzo life sciences, UK) or diluent control. Control tubes containing Calcein-AM were placed on ice. All tests were carried out in duplicate. Cells were washed twice in PBSAA at 4°c, collected using a FACS calibur (Becton Dickinson) and analysed using Cellquest software. Mean fluorescence intensity (MFI) of each sample in the FL1 channel was measured, since this corresponds to R123/BODIPY retention. The geometric mean fluorescence modulation ratio is calculated as: (MFI with modulator - cold control MFI)/(MFI with diluent control - cold control MFI) [53,54].

### Real-time PCR for ABCG2 message levels

Thawed and rested AML blasts were depleted of CD2^+ ^cells using Dynabeads (to deplete NK and T cells some of which express ABCG2 mRNA [56]. RNA from CD2 depleted AML blasts and the ABCG2 positive OCI-AML6.2 cell line was prepared using QIAamp RNA kits with DNase treatment according to the manufacturer's instructions (Qiagen). Quantitative PCR was carried out as previously reported [22]. The relative expression levels of ABCG2 transcripts were calculated as the ratio between the level of ABCG2 and the level of β2M. Both sets of real time primers used have previously been published [57,58].

### Determination of cell viability

This was done by a previously published in-house method which makes use of an internal standard and allows rapid enumeration of viable cells by flow cytometric counting of cells labelled with 7-amino Actinomycin D (7-AAD) [59].

### Histone H3 phosphorylation status

This was done by flow cytometry using anti-phospho-histone H3 (Ser 10) mouse monoclonal antibody (Upstate; now part of Millipore, Livingstone, UK) as previously described [22]. 37 primary AML samples were cultured at 10^6^/ml in RPMI 1640 with 10% FCS, 2 mM L-glutamine, supplemented with 20 ng/ml interleukin (IL)-3, 20 ng/ml stem cell factor, 20 ng/ml IL-6 + 25 ng/ml granulocyte colony-stimulating factor (R+D Systems) + 0.07 μl/ml beta-mercaptoethanol for 48 hours. Samples were then treated with 300 nM barasertib-hQPA for 60 minutes followed by pHH3 measurement by flow cytometry. pHH3 expression was calculated as a percentage of untreated controls.

### UIC2 shift assay

The reactivity of the UIC2 antibody with Pgp is increased by the addition of Pgp transported compounds and has been described by Park [44]. Briefly, cells were resuspended at 5 × 10^5^/ml in culture medium and incubated for 10 minutes at 37°C, 5% C0_2_. 1 μM barasertib-hQPA or 5 μM of the known Pgp substrate vinblastine, or both was added to cells in duplicate and incubated at 37°C, 5% C0_2 _for 4 hours. Either 0.1 μg/ml UIC2 monoclonal antibody (Immunotech) or IgG2a isotype control (Dako) was added to paired samples and incubated for a further 15 minutes. Cells were washed twice in PBSAA, resuspended in 20% Rabbit serum, and placed on ice for 30 minutes. 5 μl FITC-conjugated rabbit anti-mouse secondary antibody (Dako) was then added to cells, and following 30 minutes incubation on ice, cells were washed twice and analysed by flow cytometry. An increase in FL1 fluorescence compared to a paired control is taken to indicate the presence of a Pgp substrate.

### Radio-labelled drug accumulation assay

Pgp expressing OCI-AML3DNR cells and the Pgp null OCI-AML3 parent cell line were plated in duplicate onto 12 well plates and pre-incubated for 30 mins with or without the Pgp modulator CSA at 2.5 μg/ml. The BRCP expressing OCI-AML6.2 cells were treated similarly using BCRP null OCI-AML3 cells as a control and 10 μM FTC as the BCRP modulator. Medium containing 0.017 μCi radio-labelled [^14^C]-barasertib-hQPA (AstraZeneca, Cheshire, UK) was then added to give a final drug concentration of 300 nM. The cultures were incubated for 1 Hr at 37°c with agitation after 30 mins. The radioactive medium was then aspirated and cells washed twice with ice cold PBS before permeabilisation with 1% sodium dodecylsulphate (Sigma). Cell extracts were then added to scintillation fluid (Perkin Elmer) and radioactive drug retention measured using a TRI-CARB 2100TR liquid scintillation analyser (Packard).

### Statistical Analysis

Statistical analysis was carried out using the Statistical Package for Social Sciences, version 16 (SPSS, Chicago, IL, USA). P values of less than or equal to 0.05 were considered to represent significance.

## Competing interests

The authors declare that they have no competing interests.

## Authors' contributions

MG carried out all cell line and primary sample experiments and drafted the manuscript. MG and CS performed the RT PCR for ABCG2 message levels. CS performed the cell line genetic analysis. MP and NR conceived of the study. MP participated in the design and coordination of the study and helped to draft the manuscript. All authors critically read the manuscript and approved the final version.

## Pre-publication history

The pre-publication history for this paper can be accessed here:

http://www.biomedcentral.com/1471-2407/11/254/prepub
